# Local Epidermal Endocrine Estrogen Protects Human Melanocytes against Oxidative Stress, a Novel Insight into Vitiligo Pathology

**DOI:** 10.3390/ijms22010269

**Published:** 2020-12-29

**Authors:** Asako Yamamoto, Lingli Yang, Yasutaka Kuroda, Jiao Guo, Lanting Teng, Daisuke Tsuruta, Ichiro Katayama

**Affiliations:** 1 Department of Pigmentation Research and Therapeutics, Graduate School of Medicine, Osaka City University, Osaka 545-0051, Japan; yamamoto.asako.1021@gmail.com (A.Y.); kuroda.yasutaka@kao.com (Y.K.); guo.jiao@med.osaka-cu.ac.jp (J.G.); tenrantei@gmail.com (L.T.); katayama@derma.med.osaka-u.ac.jp (I.K.); 2 Department of Dermatology, Graduate School of Medicine, Osaka City University, Osaka 545-0051, Japan; dtsuruta@med.osaka-cu.ac.jp; 3 Biological Science Laboratories, Kao Corporation, Kanagawa 250-0002, Japan

**Keywords:** estrogen, vitiligo, oxidative stress, keratinocyte, melanocyte

## Abstract

As the outermost barrier of the body, skin is a major target of oxidative stress. In the brain, estrogen has been reported synthesized locally and protects neurons from oxidative stress. Here, we explored whether estrogen is also locally synthesized in the skin to protect from oxidative stress and whether aberrant local estrogen synthesis is involved in skin disorders. Enzymes and estrogen receptor expression in skin cells were examined first by quantitative real-time PCR and Western blot analyses. Interestingly, the estrogen synthesis enzyme was mainly localized in epidermal keratinocytes and estrogen receptors were mainly expressed in melanocytes among 13 kinds of cultured human skin cells. The most abundant estrogen synthesis enzyme expressed in the epidermis was 17β-hydroxysteroid dehydrogenase 1 (HSD17β1) localized in keratinocytes, and the most dominant estrogen receptor expressed in the epidermis was G protein-coupled estrogen receptor 1 (GPER1) in melanocytes. To investigate whether keratinocyte-derived estradiol could protect melanocytes from oxidative stress, cultured human primary epidermal melanocytes (HEMn-MPs) were treated with H_2_O_2_ in the presence or absence of 17β estradiol or co-cultured with *HSD17β1* siRNA-transfected keratinocytes. Keratinocyte-derived estradiol exhibited protective effects against H_2_O_2_-induced cell death. Further, reduced expression of HSD17β1 in the epidermis of skin from vitiligo patients was observed compared to the skin from healthy donors or in the normal portions of the skin in vitiligo patients. Our results suggest a possible new target for interventions that may be used in combination with current therapies for patients with vitiligo.

## 1. Introduction

An increased amount of the reactive oxygen species (ROS) leads to cell damage. However, our body also employs a well-regulated antioxidant mechanism to protect itself from the oxidative stress. The imbalance arising from overproduction of ROS or a deficiency in the antioxidant defense system contributes to numerous diseases.

Skin epidermal melanocytes are known to be particularly vulnerable to oxidative damage owing to the pro-oxidant state generated during melanin synthesis [1]. Vitiligo, a common skin disorder, is characterized by white patches of skin due to loss of functional epidermal melanocytes [2,3]. Oxidative stress has been discussed as a critical contributor to vitiligo pathogenesis [4,5,6]. However, the specific mechanism involved in oxidative stress-induced epidermal melanocyte disappearance is still not fully understood.

As the outermost barrier of the body, skin plays an important role in defending external and internal insults and maintaining homeostasis. It is a major candidate and target of oxidative stress. The structure of skin is composed of several layers of epidermal keratinocytes and epidermal melanocytes. Each layer is equipped with its own defense molecules. Elucidation of the mechanisms involved in skin oxidation and defense system may help to better understand various pathological processes in skin disorders.

Hormonal state has been increasingly recognized as influencing the regulation of the antioxidant defense system and maintaining homeostasis. Estrogen, a natural steroid hormone, has been known as a powerful antioxidant [7,8,9]. Recently, brain-derived local estrogen was observed, and this local synthesized hormone was proven to have the capacity to prevent neuronal cell death caused by increased oxidative burden [10,11,12,13,14,15]. Melanocytes are melanin-producing cells of the skin that are derived from neural crest cells, and in a previous study, in vitro characterization of epidermal melanocyte cultures from vitiligo revealed common features with neurodegenerative diseases [16]. Furthermore, there are accumulating data to support that the skin is not only a recipient of hormones, but is also an organized community in which cells can locally synthesize hormones [17]. Human skin is considered the largest peripheral endocrine organ [18].

Interestingly, some previous studies have demonstrated that patients with generalized vitiligo were successfully treated by oral administration of a sex hormone / thyroid powder mixture [19,20,21]. In addition, human melanocytes were previously suggested to be target cells for sex steroid beta-estradiol [22]. Given the above, we focused our attention on the local epidermal estrogen endocrine system in the skin. The purpose of our study was to investigate whether estrogen is synthesized in skin tissue and protects human melanocytes against oxidative stress and to examine whether the deficiency of the local epidermal estrogen endocrine system is involved in vitiligo pathogenesis. 

## 2. Results

### 2.1. Estrogen Synthesis Enzyme HSD17β1 Is Expressed by Epidermal Keratinocytes and Estrogen Receptor GPER1 Is Expressed Mainly by Melanocytes

**Figure 1:**
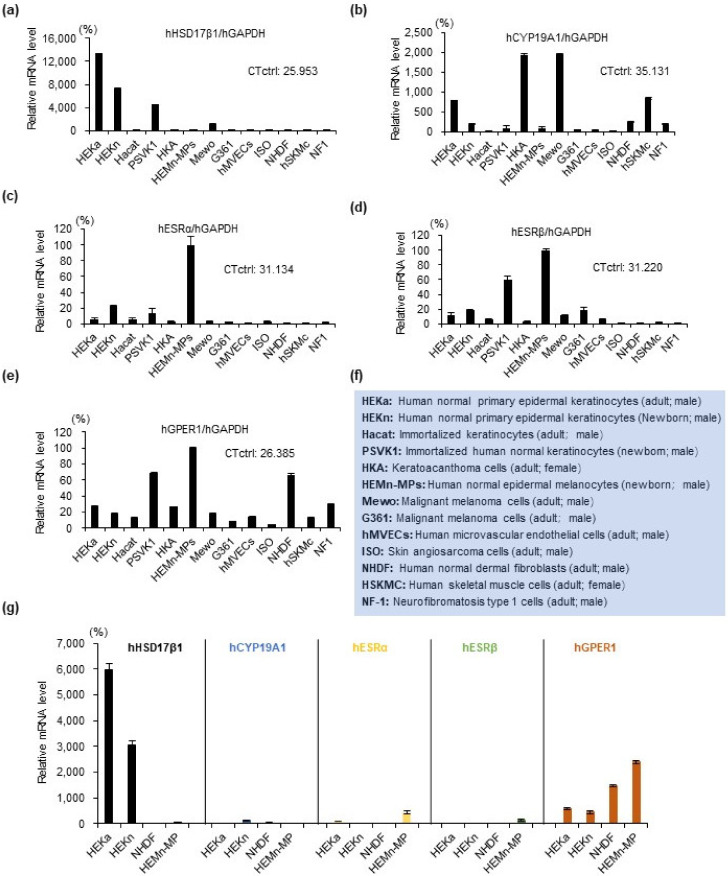
Quantitative real-time PCR was used to analyze mRNA expression levels of estrogen synthesis enzymes and receptors in 13 types of skin cells. (**a**) Relative mRNA expression level of estrogen synthesis enzyme hHSD17β1; (**b**) relative mRNA expression level of estrogen synthesis enzyme hCYP19A1; (**c**) relative mRNA expression level of estrogen receptor hESRα; (**d**) relative mRNA expression level of estrogen receptor hESRβ; (**e**) relative mRNA expression level of estrogen receptor hGPER1; (**f**) information on skin cells; (**g**) mRNA expression levels of estrogen synthesis enzymes and receptors in the indicated cell lines. mRNA expression levels were normalized to glyceraldehyde-phosphate-dehydrogenase (GAPDH). Data represent the results of three independent experiments. Data are shown as means ± SD.

We examined the expression of estrogen-related molecules in 13 types of skin cells (listed in Figure 1f) to investigate whether estrogen synthesis enzymes and receptors were expressed in skin cells and which cells expressed them. First, mRNA expression levels of estrogen synthesis enzymes (HSD17β1 and CYP19A1) (Figure 1a,b) and estrogen receptors (ESRα, ESRβ, and GPER1) (Figure 1c–e) were assessed by quantitative real-time PCR. Differential expression levels and dispersion of individual CT values of these genes in thirteen types of skin cell lines were identified. Interestingly, among the 13 kinds of cultured human skin cells, the estrogen synthesis enzyme was mainly observed in keratinocyte cell lines (HEKa, HEKn, PSVK1, Figure 1a,b), while estrogen receptors were mainly observed in melanocytes (HEMn-MPs, Figure 1c–e). Further, mRNA expression levels of these molecules were further investigated in four types of main skin cells (Figure 1g). Our data revealed that the more abundantly expressed estrogen synthesis enzyme in keratinocytes was 17β-hydroxysteroid dehydrogenase 1 (HSD17β1) (Figure 1g). The most abundantly expressed estrogen receptor among the three receptors in melanocytes was G protein-coupled estrogen receptor 1 (GPER1) (Figure 1g). 

**Figure 2:**
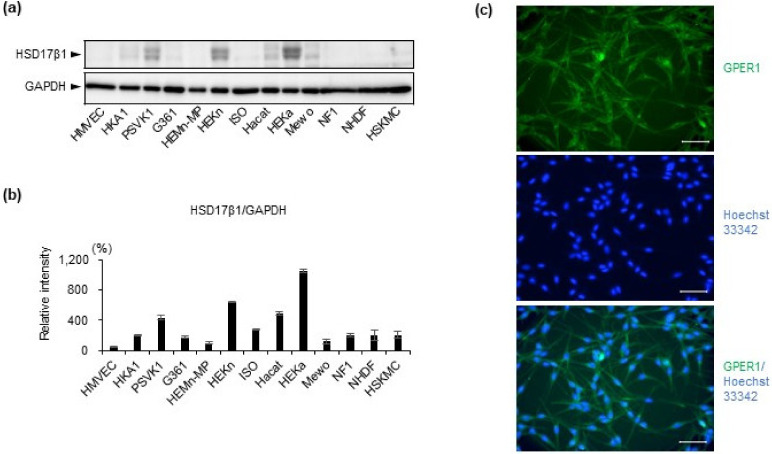
Protein expression levels of estrogen synthesis enzyme HSD17β1 and receptor GPER1 were analyzed by Western blot analyses and immunofluorescence staining. (**a**) Estrogen enzyme HSD17β1 protein expression level in lysates from indicated cultured cells was examined by Western blot analysis and the density of blot bands is quantified (**b**); (**c**) estrogen receptor GPER1 protein expression level in cultured melanocytes was examined by immunofluorescence staining with anti-GPER1 antibody (green) and Hoechst 33342 (blue). White bar, 50 µm. Data in (**b**) represent the results of three independent experiments. Data are shown as means ± SD.

Furthermore, the protein level of HSD17β1 in 13 cell lines was examined by Western blot analysis (Figure 2a,b) and had almost the same expression pattern as gene expression (Figure 1a). Highly expressed estrogen synthesis enzyme HSD17β1 was confirmed in keratinocyte cell lines (PSVK1, HEKa, and HEKn) on the protein level (Figure 2a,b). In addition, among these three keratinocyte cell lines, HEKa most highly expressed HSD17β1, both on the mRNA level and on the protein level (Figure 1a,g and Figure 2a,b). By immunofluorescence staining, protein expression of estrogen receptor GPER1 in melanocyte cell line HEMn-MPs was also confirmed (Figure 2c).

### 2.2. 17β Estradiol Does Not Increase Melanin Synthesis but Protects Melanocytes from Oxidative Stress-Induced Cytotoxicity

**Figure 3:**
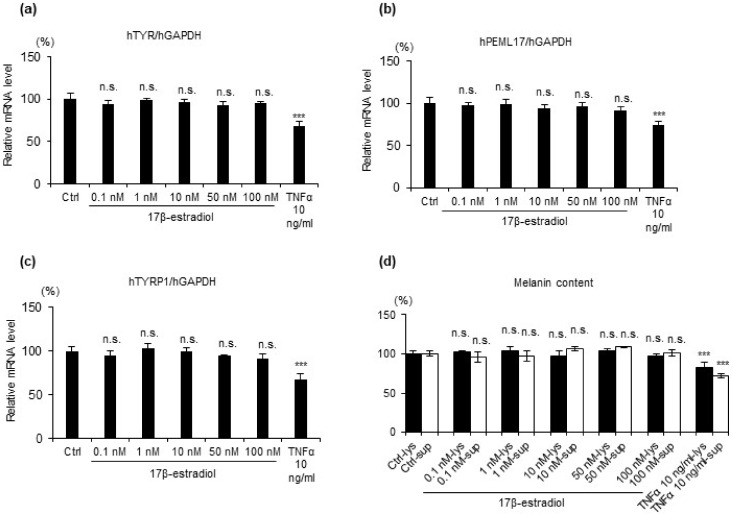
mRNA expression level of melanin synthesis-related genes and melanin content in cell lysates and culture medium after treatment with 17β-estradiol. Relative hTYR (**a**), hPMEL17 (**b**), and hTYRP1 (**c**) gene expression in cultured human epidermal melanocytes after 24 h exposure to the indicated treatments. (**b**) Melanin content quantification by melanin content assay in a culture medium; (**d**) melanin content quantification by melanin content assay in cell lysates and culture medium after 7 days exposure to the indicated treatments. Data represent the results of three independent experiments. Data are shown as means ± SD. *** *p* < 0.01; n.s., no significant difference.

Next, to clarify whether and how estrogen affects melanocytes, first, we evaluated the melanogenesis in cultured melanocytes using commercial potent estrogen agent 17β-estradiol. HEMn-MPs were stimulated with 17β-estradiol (0.1 nM, 1 nM, 10 nM, 50 nM, and 100 nM). TNFα stimulation (10 ng/mL) was used as a positive control as it is known to reduce melanin production. Gene expression level of the key enzyme in melanogenesis (Figure 3a–c) and melanin content (Figure 3d) were evaluated. After 24 h incubation with or without stimulation, cells were harvested for mRNA expression level analysis (Figure 3a–c). Seven days after stimulation, the medium and the cells were applied to melanin content measurement (Figure 3d). Compared to the control cells, the gene expression level of melanogenesis-related key enzymes *TYR*, *PMEL17*, *TYRP1* and the melanin content were markedly reduced in cells with 10 ng/mL TNFα. However, no significant change was observed in cells with 17β-estradiol.

**Figure 4:**
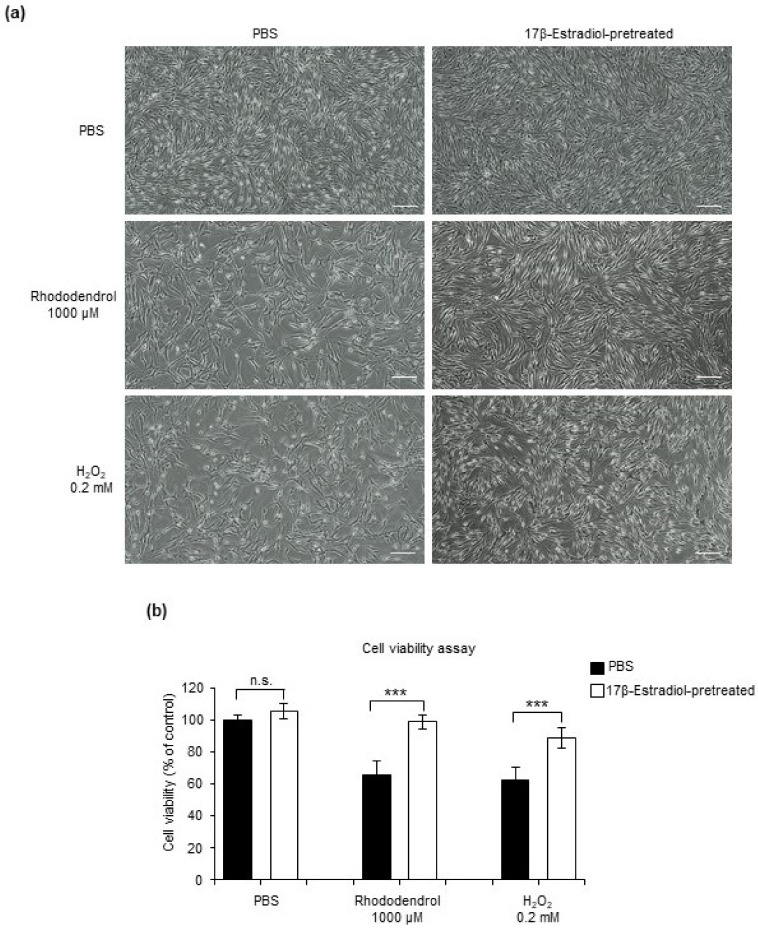
Estradiol protects melanocytes against oxidative stress. (**a**) Representative bright-field cell morphology images of cultured melanocytes with the indicated treatment. White bar, 50 µm. (**b**) Cell viability assay of cultured melanocytes with the indicated treatment. Data in **(b)** are shown as means ± SD; n.s., no significant difference; *** *p* < 0.01.

After that, cytoprotective effects were evaluated, In the present study, we used H_2_O_2_-induced and rhododendrol-induced oxidative stress in the normal human primary epidermal melanocytes as an in vitro model. HEMn-MPs were pretreated for 6 h with 50 nM 17β-estradiol and then exposed to 0.2 mM H_2_O_2_ or 1000 μM rhododendrol for 24 h. Cell viability was assessed by observing cell morphology (Figure 4a) and 3-[4,5-dimethylthiazol-2-yl]-2,5 diphenyl tetrazolium bromide (MTT) assays (Figure 4b). Morphological observations under light microscopy indicated that the exposure of melanocytes to 0.2 mM H_2_O_2_ or 1000 μM rhododendrol for 24 h resulted in obvious membrane blebbing and cell shrinkage. In contrast, 17β-estradiol pretreatment attenuated these morphological changes in melanocytes (Figure 4a). In addition to the morphological evaluation, the protective effect of 17β-estradiol against H_2_O_2_ or rhododendrol was confirmed by an MTT assay. The viabilities of 0.2 mM H_2_O_2_-treated or 1000 μM rhododendrol-treated HEMn-MPs reduced significantly compared with the control (Figure 4b); however, preincubation with 17β-estradiol for 6 h reduced both H_2_O_2_ and rhododendrol-induced cytotoxic effects on melanocytes (Figure 4a,b).

### 2.3. HSD17β1 Is Essential for Estradiol Synthesis in Keratinocytes and Keratinocyte-Derived Estradiol Attenuates Oxidative Stress-Induced Cell Death in Human Epidermal Melanocytes

**Figure 5:**
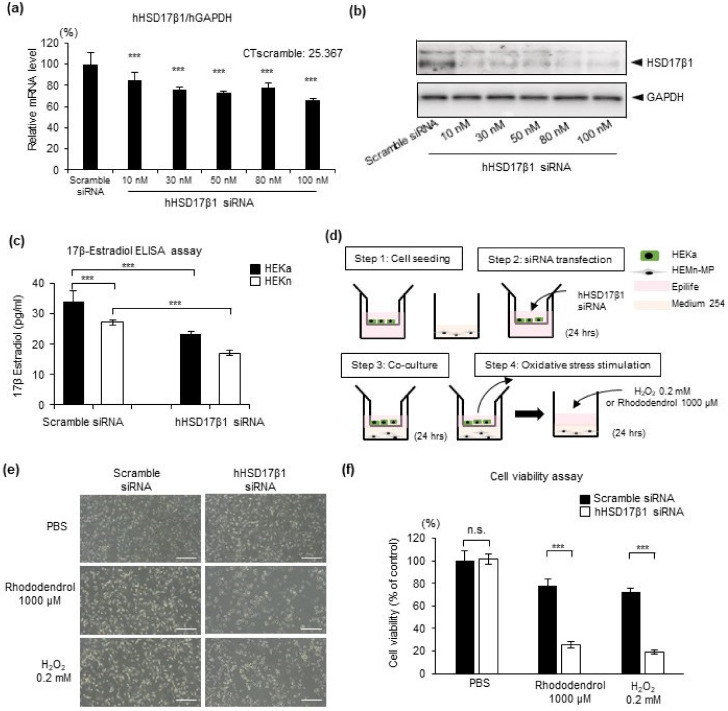
Keratinocyte-derived estradiol protects melanocytes against the oxidative stress. (**a**) In vitro, cultured human primary skin epidermal melanocytes were transiently transfected with Hhsd17β1 siRNA at the indicated concentration. Twenty-four hours after transfection, mRNA expression levels were evaluated by real-time PCR analysis. (**b**) Forty-eight hours after transfection, protein expression levels of HSD17β1 were evaluated by Western blotting. (**c**) Forty-eight hours after transfection, 17β-estradiol levels in the culture medium were evaluated by ELISA assay. (**d**) Co-culture system of HEKn and HEMn-MPs. After co-culture and the indicated treatment, cell morphology (**e**) and cell viability (**f**) were evaluated by bright microscopy and MTT assay, white bar in (**e**), 100 µm. Data represent the results of three independent experiments. Data are shown as means ± SD. *** *p* < 0.01; n.s., no significant difference.

To verify whether keratinocyte-derived estrogen has the same effect as commercial estrogen to melanocytes, the keratinocytes/melanocytes co-culture system was used. We evaluated whether the protective effect of keratinocyte-derived estrogen on melanocytes against oxidative stress is attenuated by knockdown of *HSD17β1* in keratinocytes. First, optimal transfection efficiency was gauged using a siRNA specific for HSD17β1 to transfect primary keratinocytes HEKa at various concentrations (range: 10–100 nM) and evaluating *HSD17β1* mRNA expression levels and protein levels 24 h and 48 h thereafter (Figure 5a,b). Significant inhibition of the *HSD17β1* gene and protein expression were achieved across the entire concentration gradient (Figure 5a,b). For the subsequent experiments, 30 nM siRNA was chosen. ELISA analysis helped to observe synthesis of 17β-estradiol in HEKa and HEKn (Figure 5c), and the knockdown effect was achieved in the protein expression level of 17β-estradiol 48 h after transfection (Figure 5c). It suggested that epidermal keratinocytes can locally synthesize estrogen, and enzyme HSD17β1 is essential for local estrogen biosynthesis in keratinocytes.

To clarify the effect of keratinocyte-derived estrogen on melanocytes, we developed a 4-step insert co-culture system (Figure 5d). HEKa and HEMn-MP were seeded in Epilife in insert wells and in Medium 254 in plate wells, respectively (Figure 5d, Step 1). Then, HEKa in the insert wells were transfected with 30 nM HSD17β1 siRNA (Figure 5d, Step 2). Twenty-four hours after transfection, HEKa-seeded insert wells were set up to the HEMn-MPs-seeded plate well for co-culture (Figure 5d, Step 3). After 24 h co-culture, insert wells were removed and then exposed to 0.2 mM H_2_O_2_ or 1000 μM rhododendrol for 24 h (Figure 5d, Step 4). Cell viability was assessed by observing cell morphology (Figure 5e) and MTT assays (Figure 5f). Compared to the co-culture with scramble siRNA-transfected keratinocytes, the co-culture with HSD17β1 siRNA-transfected keratinocytes markedly reduced cell viability in melanocytes under H_2_O_2_ and rhododendrol-induced oxidative stress (Figure 5e,f). It suggests that keratinocyte-derived estrogen protects melanocytes against oxidative stress.

### 2.4. Reduced HSD17β1 Expression May Be Involved in the Loss of Melanocytes in Patients with Vitiligo

**Figure 6:**
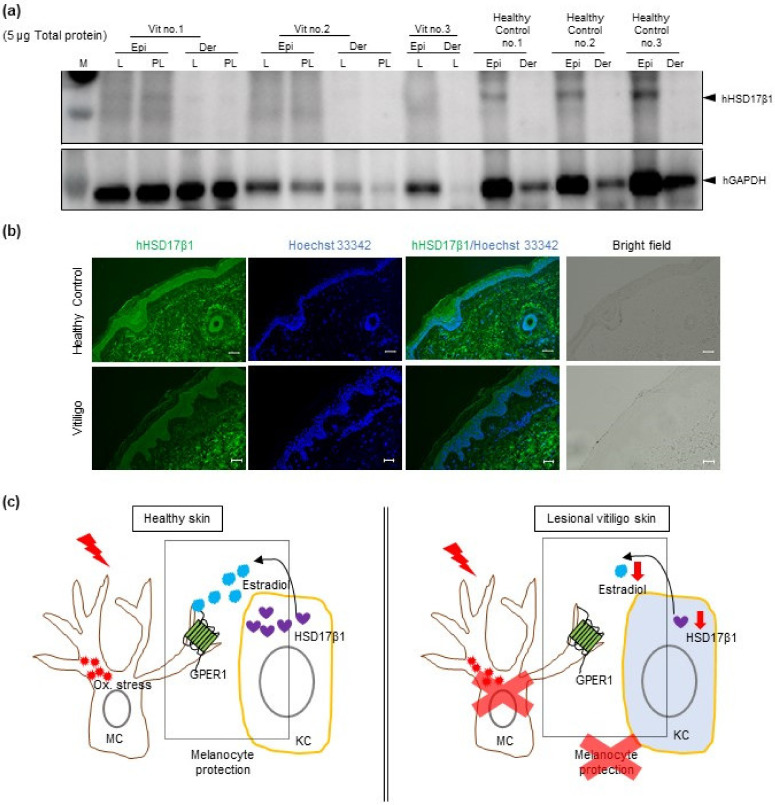
Estrogen synthesis enzyme HSD17β1 is reduced in skin epidermal keratinocytes of vitiligo patients. (**a**) Western blot analysis of 5 μg protein from vitiligo patients and healthy controls. Vit., vitiligo patient; Epi., epidermis; Der., dermis; L., lesional skin of vitiligo; PL., perilesional skin of vitiligo. (**b**) Representative sections of skin from healthy donors and vitiligo patients stained by the anti-HSD17β1 antibody (in green) and Hoechst 33342 (in blue) are shown. The corresponding bright-field microscopy image is shown in the lower panel. White bar, 100 µm. (**c**) Summary illustration of the possible involvement of local estrogen in vitiligo development. KC, keratinocytes; MC, melanocytes.

Previous studies revealed that oxidative stress plays a critical role in melanocyte apoptosis and was considered to be a major cause of vitiligo [1]. We then sought to determine whether estrogen was involved in the pathogenesis of vitiligo. The expression of HSD17β1 was evaluated by Western blot analysis using 5 μg of protein extracts from the epidermis and dermis of lesional or perilesional vitiligo skin (*n* = 3) or healthy control skin (*n* = 3) (Figure 6a). Interestingly, our data show that HSD17β1 is mainly expressed in the epidermis (Figure 6a), which is consistent with our data that HSD17β1 is mainly expressed in epidermal keratinocytes (Figure 1). Further, compared to healthy controls, the expression of HSD17β1 is remarkably reduced in the epidermis of vitiligo patients, both in the lesional and perilesional skin (Figure 6a).

In addition, HSD17β1 expression was also examined by histo-immunofluorescence staining with an antibody to HSD17β1. Lesional skin from confirmed vitiligo patients in progressive states (*n* = 7) and samples from corresponding sites of healthy donors (*n* = 4) were analyzed (Figure 6b). Compared to the skin from healthy controls, reduced expression of HSD17β1 in the epidermal keratinocytes of vitiligo patients was also observed (Figure 6b).

As shown in the illustration (Figure 6c), these results demonstrated that estradiol in healthy skin was constantly converted and produced by estrogen enzyme HSD17β1 in local skin keratinocytes and released into the extracellular space between keratinocytes and melanocytes in the epidermis. This then protects melanocytes against oxidative stress and maintains the redox balance for melanocytes in basal conditions (Figure 6c, left panel). However, in the skin of patients with progressive vitiligo, oxidative stress or intrinsic defects induce lower HSD17β1 expression in keratinocytes leading to the failure of keratinocytes in producing sufficient estradiol to protect melanocytes against oxidative stress, finally contributing to the destruction of melanocytes and vitiligo development (Figure 6c, right panel).

## 3. Discussion

In this study, we demonstrated that estrogen was locally synthesized in epidermal keratinocytes. Further, keratinocyte-derived estrogen attenuated oxidative stress-induced cell damage in human epidermal melanocytes. Interestingly, decreased local estrogen synthesis enzyme expression was observed in the lesional skin of vitiligo. These findings point toward novel concepts in our understanding of the role of estrogen in skin homeostasis and vitiligo pathogenesis.

In the current study, interestingly, among the 13 kinds of cultured human skin cells, estrogen synthesis enzyme was mainly observed in keratinocyte cell lines (HEKa, HEKn, PSVK1, Figure 1 and Figure 2), while estrogen receptors were mainly observed in melanocytes (HEMn-MPs, Figure 1 and Figure 2). Our data revealed that the more abundantly expressed estrogen synthesis enzyme in keratinocytes was 17β-hydroxysteroid dehydrogenase 1 (HSD17β1) (Figure 1 and Figure 2). The most abundantly expressed estrogen receptor among the three receptors was G protein-coupled estrogen receptor 1 (GPER1) (Figure 1 and Figure 2). Furthermore, protein levels of HSD17β1 in the 13 cell lines were examined by Western blot analysis (Figure 2) and exhibited almost the same gene expression pattern. Our present observation revealed that HSD17β1 expression was robustly decreased in the lesional skin of vitiligo patients in progressive states when compared to healthy control skin or normal skin samples from vitiligo patients (Figure 6).

Estrogen, a member of the steroid hormone family and a well-recognized sex hormone, is known to be primarily produced by ovaries and testes and mainly has a critical role in regulating the development and function of male and female reproductive organs. However, recently, ever-growing evidence demonstrates that estrogen is also synthesized and released from extragonadal organs, including the adrenal gland, brain, adipose tissue, pancreas, and skin, and that it also exerts various roles in non-reproductive organs [17,23,24,25]. Previously, for a long time, it has been believed that estrogen may contribute to the depigmentation process of vitiligo, because the initiation or progression of vitiligo is observed at pregnancy, postpartum, menopause, or after using contraceptives. However, until now, there are still no sufficient scientific experimental data to explain or support this hypothesis. As is well known, pregnancy, postpartum, menopause, and the use of contraceptives may also induce psychological stress, which is known to exacerbate vitiligo. Furthermore, with regard to skin, the function of systemic estrogen may be quite different from that of local synthesized estrogen. 

In addition, there are some other studies showing that estrogen triggers generation of H_2_O_2_ and contributes to DNA damage in human peripheral blood lymphocytes; however, results are quite controversial. In one study, DNA damage was observed in normal peripheral blood lymphocytes [26]. However, in another study, DNA damage was only observed in peripheral blood lymphocytes of patients with vitiligo, but not in that of healthy controls [27]. The effects of estrogen on epidermal melanocytes were not documented. 

In the present study, we first clarified local estrogen biosynthesis by keratinocytes in the epidermis and the novel protective effect of extragonadal estradiol on melanocyte dysfunction under oxidative stress. Interestingly, consistent with our results, some previous studies demonstrated that patients with generalized vitiligo were successfully treated by oral administration of a female hormone powder mixture [19,20,21]. Presently, the exact mechanism of estrogen’s protective effects against oxidative stress remains to be elucidated in our future study. However, it has been reported that the observation that the elevated expression of biological markers in cells from neurodegenerative diseases such as Alzheimer’s can also be detected in vitiligo melanocytes [16]. Local estradiol in skin might exert protective effects equal to those observed in the brain [10].

In conclusion, our data demonstrated that local estradiol production exists in the skin and plays an important antioxidant role in protecting melanocytes from oxidative stress. More importantly, the dysregulation of skin-derived estrogen synthesis contributes to enhanced sensitivity of vitiligo melanocytes to oxidative stress in the epidermis. Our findings might indicate a novel insight into the pathogenic mechanism of vitiligo and provide prospective therapeutic interventions which target estrogen signaling for vitiligo prevention or treatment.

## 4. Materials and Methods

### 4.1. Cell Lines and Cell Culture

Normal neonatal human epidermal keratinocytes (HEKn) and normal adult human epidermal keratinocytes (HEKa) were purchased from Cascade Biologics™ (Portland, OR, USA), and cultured in the Epilife medium supplemented with Human Keratinocyte Growth Supplement (HKGS) (GIBCO, Gaithersburg, MD, USA). Human immortalized keratinocytes (HaCaT cells) were purchased from the American Type Culture Collection (ATCC, Manassas, VA, USA), human keratinocytes PSVK1 were purchased from the Japanese Collection of Research Bioresources (JCRB, Osaka, Japan) and cultured in the KBM-Gold Keratinocyte Basal Medium (Lonza, Walkersville, MD, USA) supplemented with the KGM-Gold Bullet Kit (Lonza). Neonatal foreskin primary human epidermal melanocytes (HEMn-MP) were purchased from Cascade Biologics™ and cultured in Medium 254 (Cascade Biologics™) with the human melanocyte growth supplement (HMGS) (Cascade Biologics™). The cell line derived from a keratoacanthoma of human skin (HKA) and human melanoma cell lines Mewo and G361 were purchased from the Japanese Collection of Research Bioresources (JCRB, Osaka, Japan) and cultured in a high-glucose Dulbecco’s modified Eagle’s medium (DMEM) supplemented with 10% fetal bovine serum and 1% penicillin–streptomycin (ThermoFisher, Waltham, MA, USA). Human microvascular endothelial cells were purchased from Lonza (Walkersville, MD, USA) and cultured in the endothelial basal medium-2 (EBM-2, Lonza). Human neurofibromatosis cell line NF1 was purchased from the Japanese Collection of Research Bioresources (JCRB, Osaka, Japan) and cultured in the RPMI 1640 medium (GIBCO) with 20% fetal bovine serum (ThermoFischer). Human Muscle Basal Medium was supplemented with the human epidermal growth factor, fetuin, bovine serum albumin, dexamethasone, insulin, and he gentamicin/amphotericin B solution (all from Lonza). Skin angiosarcoma cell line ISO-HAS-B (ISO) was purchased from the Cell Resource Center for Biomedical Research of the Cell Bank (Sendai, Japan) and cultured in a high-glucose Dulbecco’s modified Eagle’s medium (DMEM) supplemented with 10% fetal bovine serum and 1% penicillin–streptomycin (ThermoFisher). Sources of all the cultured cells (e.g., gender and age generation) are listed in Figure 1f. All cultures were maintained at 37 °C in an incubator with 5% CO_2_ in the air.

### 4.2. Human Skin Specimens

Three-millimeter punch fresh skin biopsies from patients with confirmed vitiligo in the progressive state (*n* = 3) and samples from corresponding sites of healthy donors (*n* = 3) were used for protein extraction. Paraffin-embedded tissue sections of lesional skin from confirmed vitiligo patients in the progressive state (*n* = 7) and samples from corresponding sites of healthy donors (*n* = 4) were used in histo-immunofluorescence staining. Written informed consent was obtained from all participants prior to study inclusion. The study was approved by the ethics committee of the Osaka City University Faculty of Medicine (No. 4152).

### 4.3. Cell Treatment

Twenty-four hours after cell seeding, HEKa, HEKn, and HEMn-MPs were treated with H_2_O_2_ (0.2 mM), rhododendrol (1000 μM), or estradiol (25 nM) for the indicated periods prior to RNA and protein extraction. HEKa and HEKn were used during the third passage, while HEMn-MPs were used during the twelfth passage. In each experiment, cultured cells were treated at the same time and under the same culture conditions (e.g., cell density, passage, and days after plating). For co-culture, HEKa and HEMn-MPs were grown in insert wells (Corning) and plate wells (Corning), respectively, and the medium consisted of Epilife and Medium 254 (1:1).

### 4.4. RNA Isolation and Quantitative RT-PCR Analysis

Total RNA was extracted from cultured cells using a Maxwell^®^ 16 LEV simplyRNA Tissue Kit (Promega, Netherlands) following the manufacturer’s instructions. Total RNA (100 ng) was reverse-transcribed using the ReverTra Ace qPCR RT Master Mix (TOYOBO, Osaka, Japan).

The primers used for real-time PCR were as follows:

Primers used for real-time PCRGene NameSenseAntisensehHSD17b15′-TTATTGCGCCAGCAAGTTCG-3′5′-TTCTCCATGAAGGCGGTGTG-3′hCYP19A15′-CGGCCTTGTTCGTATGGTCA-3′5′-CAGAAGGGTCAACACGTCCA-3′hESRα5′-GGAGAGGAGTTTGTGTGCCT-3′5′-CCAGGACTCGGTGGATATGG3′hESRβ5′-CGTGACCGATGCTTTGGTTT-3′5′-AGCAGATGTTCCATGCCCTT-3′hGPER15′-CTGCTTCTGTTTCGCGGATG-3′5′-ACCCGGACAATGAGGGAGTA-3′hTYR5′-TGACTCCAATTAGCCAGTTCCT-3′5′-GACAGCATTCCTTCTCCATCAG-3′hPMEL175′-CTATGTGCCTCTTGCTCATTCC-3′5′-TGCTTGTTCCCTCCATCCA-3′hTYRP15′-CTCAATGGCGAGTGGTCTGT-3′5′-TTCCAAGCACTGAGCGACAT-3′hGAPDH5′-GACAGTCAGCCGCATCTTCT-3′5′-GCGCCCAATACGACCAAATC-3′

Real-time PCR was carried out using a QuantStudio^®^5 Real-time PCR System (Applied Biosystems, San Francisco, CA, USA). Reactions were run in triplicate during three independent experiments. The geometric mean of the housekeeping gene, GAPDH, was used as an internal control to normalize variability in expression levels.

### 4.5. Melanin Content Assay

To determine melanin content, cells were dissolved in 200 μL of 1 N NaOH for 30 min at 100 °C to solubilize the melanin, which was then quantified in cell suspensions by recording the absorbance at 405 nm as described previously [28]. Melanin content was calculated and corrected based on cell number.

### 4.6. Cell Viability Assay

HEMn-MPs (1 × 10^4^ cells/well) were cultured in 96-well flat-bottom tissue culture plates. After experimental treatments, cells were washed three times with cold PBS, and cell viability was determined using the Cell Count Reagent Survival Fraction (SF) colorimetric assay (Nacalai Tesque, Kyoto, Japan) according to the manufacturer’s instructions.

### 4.7. Western Blot Analysis

For protein sample preparation, cell pellets were extracted as described previously [28] and 5 µg of the extracted protein were used for Western blot analysis. The primary antibodies were used at the following dilutions: anti-HSD17β1 (Abcam, Cambridge, MA, USA) at 1:1000 and anti-GAPDH (CST, Danvers, MA, USA) at 1:1000. We used the anti-GAPDH antibody as a loading control.

### 4.8. Immunofluorescence Staining of Cells

HEMn-MPs were seeded on six-well plates precoated and grown to confluence. Twenty-four hours after seeding, cells were washed with ice-cold PBS and fixed with 4% paraformaldehyde for 5 min. Cells were stained with anti-GPER1 (HPA027052, Sigma-Aldrich, St. Louis, MO, USA) at a ratio of 1:50 followed by incubation with a secondary antibody (anti-rabbit IgG Alex Fluor 488, 1:500 dilution, Invitrogen, Carlsbad, CA, USA). Hoechst 33342 (Invitrogen, 1:500 dilution) was used to stain nuclei. Cells were visualized using a Biozero 8100 confocal microscope (Keyence Corporation, Osaka, Japan).

### 4.9. Fluorescent Immunohistochemistry Staining

Skin tissue samples were fixed in 10% formaldehyde and embedded in paraffin. Then, 3 μm sections were used for fluorescent immunohistochemistry staining. Sections were incubated with the primary antibodies specific for HSD17β1 (1:100 dilution, Abcam) overnight at 4 °C and then incubated with a secondary antibody (anti-rabbit IgG AlexFluor 488, Invitrogen) [29]. Sections were counterstained with Hoechst 33342 at a ratio of 1:500 (Invitrogen). The stained sections were visualized using a light microscope or a Biozero 8100 confocal microscope (Keyence).

### 4.10. RNA Interference

For small interfering RNA (siRNA)-mediated knockdown of hHSD17β1, human HSD17β1 siRNA (ID 112609) and negative control siRNA were purchased from ThermoFischer. Cells were then transfected with 10~100 nM of either the target siRNA or the control siRNA using Lipofectamine RNAi (Invitrogen) for 48~96 h. The functional assays were subsequently done after validation of hHSD17β1 knockdown without affecting cell viability.

### 4.11. ELISA Assay

The HEKa and HEKn culture supernatants were collected and the concentration of 17β-estradiol was measured using an ELISA kit for 17β-estradiol (ab108640, Abcam) according to the manufacturer’s instructions. The absorbance values at 450 nm were measured by a Model 680 microplate reader (Bio-Rad Laboratories, Hercules, CA, USA).

### 4.12. Statistical Analysis

Experiments were repeated at least three times. Data are presented as means ± standard deviation (SD). Statistical analysis was conducted using two-way analysis of variance for interactions between variance with Tukey’s or Bonferroni post-hoc tests. Unpaired Student’s *t*-test (Microsoft Excel; Microsoft Corp., Redmond, WA, USA) was used for comparison between groups. *p*-values < 0.05 were considered statistically significant.

## Data Availability

No new data were created or analyzed in this study.
